# Pylephlebitis: A Case of Inferior Mesenteric Vein Thrombophlebitis in a Patient with Acute Sigmoid Diverticulitis—A Case Report and Clinical Management Review

**DOI:** 10.1155/2019/5341281

**Published:** 2019-01-21

**Authors:** Aleena Zia, Sumit Sohal, Chris Costas

**Affiliations:** ^1^Department of Internal Medicine, Presence Saint Francis Hospital, 355 Ridge Avenue, Evanston, IL 60202, USA; ^2^Department of Infectious Diseases, Presence Saint Francis Hospital, 355 Ridge Avenue, Evanston, IL 60202, USA

## Abstract

Pylephlebitis is a rare complication of intra-abdominal infections and describes thrombosis and infection as two different pathophysiological phenomena in the cause of this disease. The nonspecific presentation of disease makes its diagnosis difficult and thus leads to high mortality. The treatment comprises antibiotics and also includes controversial use of anticoagulation in these patients. Here, we present a patient with past medical history of human immunodeficiency virus and past diverticulitis who presented with fever, chills, diarrhea, neck pain, and photophobia. He was diagnosed with acute sigmoid diverticulitis with associated inferior mesenteric vein thrombophlebitis. He improved after intravenous antibiotics and anticoagulation and was discharged. He underwent sigmoid colectomy 3 months after his initial presentation and was advised to take anticoagulation for a total of 6 months.

## 1. Introduction

Pylephlebitis is defined as a suppurative thrombosis of the portal mesenteric venous system that is associated with intra-abdominal infections [1]. Although a rare phenomenon, it is associated with high mortality rates [2, 3]. Clinical presentation of pylephlebitis may be highly variable and usually requires a high clinical suspicion for diagnosis, but early and aggressive interventions play an important role in predicting the outcomes of the disease process.

## 2. Case Presentation

The patient is a 48-year-old male with past medical history of human immunodeficiency virus (HIV) on highly active antiretroviral therapy (HAART) and diverticulitis who presented to the ED with fevers and chills for 1 week associated with diarrhea, head and neck pain, and photophobia. He denied any abdominal pain and stated that his diarrhea had resolved by the time he was seen. His highest temperature at home was 104°F. He has had 2 episodes of acute diverticulitis in the past 2 years, in which he complained of left lower quadrant pain associated with nausea and vomiting. He stated that this current episode was unlike the past.

On physical examination, the patient had a temperature of 99.3°F, heart rate of 110, blood pressure of 148/84 mm Hg, and breathing at a rate of 16 with 95% oxygen saturation on room air. He was completely alert and oriented, with no neck stiffness. He had tenderness to palpation in the left upper quadrant and in the periumbilical area. Rest of his exam was essentially normal. His laboratory data revealed a white blood count of 11.4 k/cu·mm (reference range: 4.0–11.0 k/cu·mm), hemoglobin of 15.7 g/dl (reference range: 13.0–17.0 g/dl), glucose of 102 mg/dl(reference range: 70–99 mg/dl), sodium of 131 mmol/L(reference range: 133–144 mmol/L), chloride of 97 mmol/L (reference range: 98–107 mmol/L), and ALT of 56 IU/L (7.0–52.0 IU/L). The rest of the laboratory data was within normal limits.

**Figure 1:**
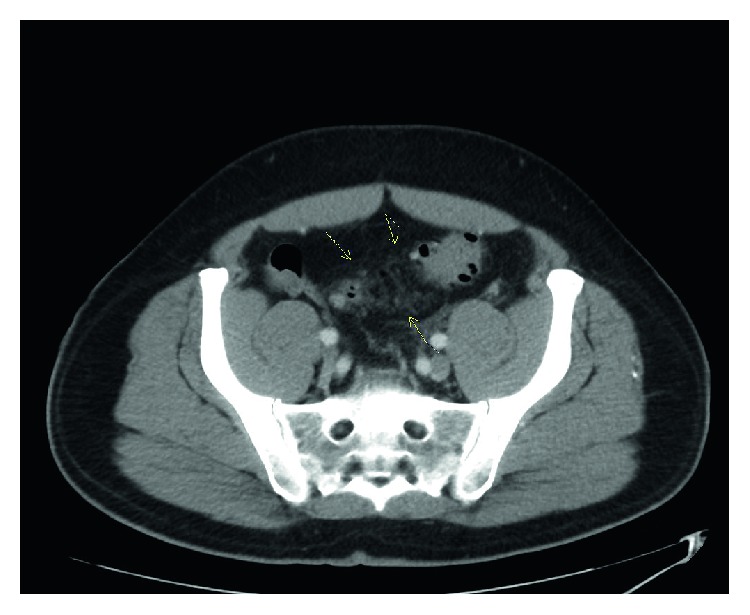
CT abdomen showing pericolonic induration (marked with arrows).

**Figure 2:**
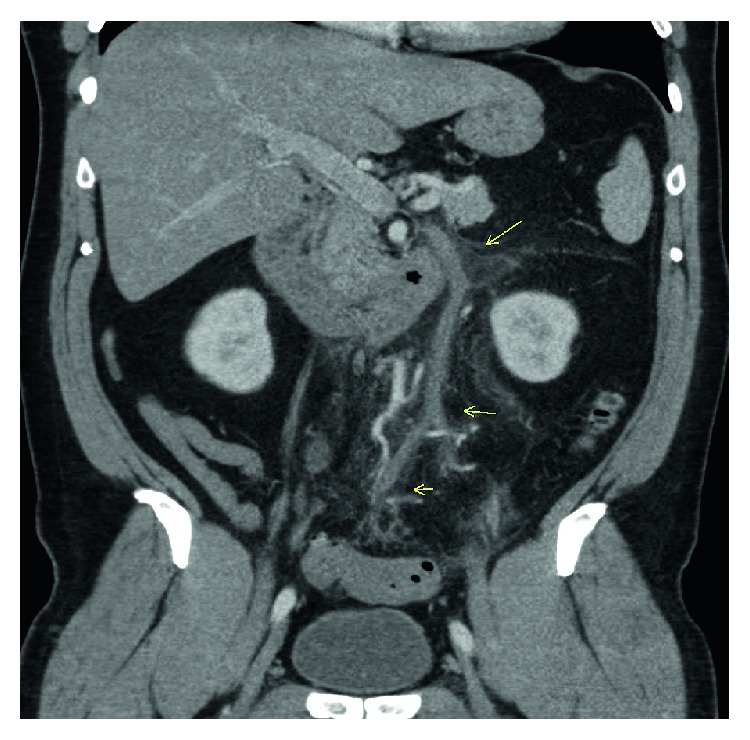
CT abdomen showing induration of the mesentery along the entire course of the inferior mesenteric vein with partial filling defects (marked with arrows).

The patient underwent a computed tomography (CT) head, and lumbar punctures which were negative for meningitis. He then underwent a CT of his abdomen and pelvis in the emergency department which showed wall thickening of the sigmoid colon with pericolonic induration suggestive of acute sigmoid diverticulitis (Figure 1). On further review, CT abdomen also showed haziness/induration of the mesentery along the entire course of the inferior mesenteric vein with partial filling defects throughout its course suggestive of inferior mesenteric vein thrombophlebitis (Figure 2). The patient was admitted and infectious disease, general surgery, and gastroenterology followed up the patient while in the hospital and was on intravenous cefoxitin and metronidazole. His blood cultures were positive for *E. coli* resistant to ciprofloxacin. His condition improved and was transitioned to oral cephalexin. He was started on oral anticoagulation with rivaroxaban and discharged with outpatient follow-up with infectious disease and general surgery.

On follow-up, the patient did not report any abdominal symptoms with no reported episodes of bleeding. Given multiple episodes of diverticulitis, the patient opted for surgical intervention and underwent laparoscopic sigmoid colon resection 3 months later. The surgical specimen reported multiple diverticula without any gross abnormalities. The patient continued to take anticoagulation medication for a duration of 6 months and did not report any abdominal symptoms or adverse events. A repeat CT abdomen was not done at the end of his course as the patient did not report any symptoms, and there is no current evidence to support repeat imaging to document recanalization. The patient underwent right upper quadrant ultrasound 1 year after the surgery for elevated liver enzymes which showed increased echogenicity within the liver but no focal abnormalities, a patent portal vein, no duct dilation, and no evidence of extension of thrombus.

## 3. Discussion

Pylephlebitis was first described in 1846 by Waller who discovered it as the source of a hepatic abscess during autopsy [4]. The true incidence of pylephlebitis is unclear, but most recent studies have actively identified this disease in the setting of availability of advanced imaging techniques [5].

### 3.1. Anatomy and Pathophysiology

Pylephlebitis has a complex pathophysiology and describes two different insults at the same time leading to its diagnosis. It is a portal mesentric venous thrombosis (PMVT)that occurs in the background of an infectious process. The real diagnosis relies on the demonstration of PMVT in the setting of suppurative bacteremia through aspiration of the portal vein; however, given the invasiveness of the process, proxy indicators of infection are used, which however are not always detectable [6].

Pylephlebitis begins with thrombophlebitis of the small veins draining the infected site. The involvement, by extension, of larger veins leads to septic thrombophlebitis of the larger mesenteric veins or portal vein [7]. A systematic review of the literature revealed that infections in general and more specifically HIV (e.g., our patient) increase the risk of venous thrombosis [8], although other factors such as coagulation disorders, portal hypertension (e.g., due to cirrhosis), malignancy, postoperative dehydration, sepsis, and trauma may complicate the process [9].

In a 1948 report, the most common inciting infection was appendicitis, accounting for all 21 cases [10]; however, more recently, diverticulitis and other intra-abdominal infections have taken over. These may include diverticulitis (30%, mostly sigmoid), appendicitis (19%), inflammatory bowel disease (6%), pancreatitis (5%), infectious enteritis (4%), bowel perforation, and malignancies (6%) [9].

The portal vein arises from the confluence of the superior mesenteric, inferior mesenteric, and splenic vein posterior to the neck of the pancreas. The portal trunk divides into two branches in the liver hilum: left portal vein branch (LPV) and right portal vein branch (RPV) [11]. The portal vein drains the abdominal part of the digestive tube (excluding the lower anal canal but including the preterminal esophagus), the spleen, pancreas, and gallbladder, and thus, pylephlebitis may occur from infection from any of these parts of the abdomen [12]. A study of 95 patients identified the right portal vein as the most common (33%) site of thrombosis, whereas the inferior mesenteric vein was the least common site (8%) among these patients [6].

Our patient had diverticulitis and later was diagnosed with pylephlebitis with involvement of the inferior mesenteric vein which is one of the least common sites of involvement.

### 3.2. Clinical Feature and Complications

Symptoms of pylephlebitis are often nonspecific; however, abdominal pain and fever are the most common findings at presentation [3, 5, 13]. Other nonspecific clinical features include fatigue, malaise, chills, nausea, vomiting diarrhea, and anorexia and weight loss [1, 14]. More advanced signs include hepatomegaly and jaundice.

Bowel ischemia and infarction may occur due to superior mesenteric vein thrombosis, and hepatic abscesses may complicate portal vein thrombophlebitis [15–17].

The clinical manifestations are often confusing and nonspecific, thus usually require a high clinical suspicion for diagnosis.

Our patient was admitted with abdominal pain and fever, which as per various studies are the most common presenting features; however, at the same time, they are very nonspecific and associated with underlying abdominal infection.

### 3.3. Diagnosis

Lab testing is very nonspecific. Leukocytosis may be a common early finding, but both normal and decreased white blood cell counts have been noted in the literature [6]. Liver function test abnormalities may or may not be present. In one study, positive blood cultures were found to be positive in 44% of patients [6], whereas other studies have shown the rate to be between 50 and 88% [1]. Infection in pylephlebitis is commonly polymicrobial, but when a single microorganism is cultured, it is most frequently *B. fragilis.* However, organisms such as *Escherichia coli*, *Proteus mirabilis*, *Clostridium sps*, and *Streptococcus species* have also been associated [1, 6, 7].

The diagnosis of pylephlebitis is primarily made by radiological means. Doppler ultrasound and contrast-enhanced CT facilitate early diagnosis. Ultrasound may show portal vein thrombosis, and contrast-enhanced CT scan can display intra-abdominal processes like appendicitis and diverticulitis as well as mesenteric and portal vein thrombosis, liver abscesses, and bowel ischemia [15].

In our patient, the CT of the abdomen not only showed acute sigmoid diverticulitis but also showed inferior mesenteric vein thrombophlebitis. Our patient also had blood cultures drawn, and they grew *Escherichia coli*.

### 3.4. Treatment

#### 3.4.1. Antibiotics

Antibiotics are the mainstay of treatment in pylephlebitis irrespective of the blood culture results. The choice of antibiotics is constituted in such a way that both Gram-negative and anaerobic infections are covered. A standard regimen and duration of antibiotic therapy has not been established; however, metronidazole, gentamicin, piperacillin, ceftizoxime, imipenem, and ampicillin have been used with success and should be given for 4 weeks to patients without liver abscess and for 6 weeks to those with liver abscess [1, 3, 18].

Our patient was given a total of 30 days of antibiotic therapy and was discharged on cephalexin.

#### 3.4.2. Anticoagulation

The most controversial topic of discussion in pylephlebitis is about anticoagulation. Some studies such as those of Plemmons et al. [19] and Kanellopoulou et al. [20] indicate the use of anticoagulation, whereas Baril et al. [21] suggest judicious use of anticoagulation.

Plemmons et al. noted a 100% survival rate among patients who received anticoagulation with heparin compared to 60% survival among those who were not anticoagulated, although statistical significance was not reached. Kanellopoulou et al. noted that the early use of anticoagulation in portal vein thrombosis may minimize serious sequelae and speed up recanalization. Baril et al. suggested that anticoagulation may only be required in patients with intrinsic hypercoagulable state or extensive thrombosis.

Our patient was started on novel oral anticoagulants (NOACs) therapy and was advised to continue for a total duration of 6 months. There are no trials available to indicate a specific anticoagulation therapy; however, warfarin, heparin, and low-molecular-weight heparin have been used in the past with recent increase in use of NOACs in sporadic case reports [22, 23]. Off-label use of rivaroxaban has been reported in cases with splanchnic venous thrombosis [24]. Similarly, there are no trials on duration of anticoagulation therapy but most of the evidence supports 3–6 months of therapy in the absence of thrombotic disease [6, 22, 23].

#### 3.4.3. Surgical Methods

Surgery may be required to remove the source of infection. Liver abscess depending upon size may also require drainage. The role of thrombolytics has been very limited and is based on few case reports. Sherigar et al. and Tamaki et al. used thrombolytics in their treatment of pylephlebitis; however, no consensus has been made on their use in this disease [25, 26].

## 4. Conclusion

This case calls for increased awareness of the complication of pylephlebitis in patients admitted with intra-abdominal infections. As the mortality rates can be higher with complication, early diagnosis and management can reduce mortality in these patients. Although anticoagulation has been a topic of controversy for these patients, our patient who received novel anticoagulation therapy did not encounter any problems with the therapy and had a good follow-up for 6 months on anticoagulation with no major adverse events reported.
